# Late-onset spontaneous chorioamniotic membrane separation mimicking amniotic band sequence with rapid progression to preterm premature rupture of membranes

**DOI:** 10.1515/crpm-2026-0024

**Published:** 2026-10-22

**Authors:** Cem Terece, Tuğban Seçkin Keçecioğlu, Simge Tezel Yozgat, Mehmet Keçecioğlu

**Affiliations:** Department of Obstetrics and Gynecology, Balikesir Ataturk City Hospital, Balikesir, Türkiye; Department of Gynecologic Oncology, Izmir City Hospital, Izmir, Türkiye

**Keywords:** chorioamniotic membrane separation, amniotic band sequence, fetal macrosomia, preterm premature rupture of membranes, third trimester

## Abstract

**Objectives:**

Spontaneous chorioamniotic membrane separation (CMS) diagnosed in the third trimester is rare, and its short-term clinical course remains poorly characterized. We report a case of late-onset CMS with rapid progression to preterm premature rupture of membranes (PPROM).

**Case presentation:**

A 23-year-old woman with a prior cesarean delivery was referred at 35 weeks for suspected fetal macrosomia. At 36+4 weeks, ultrasonography revealed freely mobile, multilayered membranes adjacent to the fetal extremities and umbilical cord, raising concern for amniotic band sequence. Detailed anatomical assessment and color Doppler evaluation demonstrated intact extremities and preserved distal perfusion, supporting a diagnosis of CMS. The patient was admitted for surveillance, and PPROM occurred within hours of diagnosis. Cesarean delivery resulted in a live male infant weighing 4,170 g (Apgar scores 8/9). Intraoperative findings confirmed near-complete amnion–chorion separation.

**Conclusions:**

Late-onset spontaneous CMS may mimic amniotic band sequence and can progress rapidly to membrane rupture. Careful sonographic differentiation is essential. Given the potential for rapid clinical deterioration, close surveillance may be warranted, particularly in late gestation.

## Introduction

Chorioamniotic membrane separation (CMS) identified beyond the expected timing of amnion–chorion fusion represents a pathological condition with heterogeneous clinical implications [1], 2]. While most reported cases are associated with invasive intrauterine procedures, spontaneous CMS remains rare and poorly characterized, particularly with respect to its short-term clinical behavior and risk of acute complications [3], 4].

Reported adverse perinatal outcomes include preterm birth, preterm premature rupture of membranes (PPROM), umbilical cord complications, and amniotic band formation, although the available evidence is largely limited to isolated case reports and small case series [2], 3]. Most diagnoses are made in the second trimester, often incidentally or in the context of prior intervention [3], 5].

In contrast, third-trimester spontaneous CMS presenting with acute onset and freely mobile membrane structures is rare and may represent a clinically urgent condition. Its sonographic appearance may closely mimic amniotic band sequence (ABS), an entity associated with a direct risk of fetal injury, including soft-tissue constriction and amputation [6]. Accurate differentiation between these entities is therefore essential due to their distinct clinical implications [1], 3], 7].

We report a case of late-onset spontaneous CMS in a macrosomic pregnancy, presenting as a sonographic mimic of ABS and progressing to PPROM within 12 h of diagnosis. This case highlights that late-onset CMS may represent a time-sensitive condition with potential for rapid deterioration, underscoring the importance of prompt recognition and appropriate surveillance.

## Case presentation

A 23-year-old woman, gravida 2 para 1, with a history of prior cesarean delivery for fetal distress, was referred to our tertiary referral center at 35 weeks of gestation for evaluation of suspected fetal macrosomia. First-trimester combined screening had indicated low aneuploidy risk, and second-trimester anomaly scanning had revealed no major structural abnormalities. A 75-g oral glucose tolerance test performed during the index pregnancy returned values within normal limits at all time points, excluding gestational diabetes as a contributory etiology for accelerated fetal growth.

Ultrasonographic evaluation at 35 weeks confirmed fetal abdominal circumference (AC) and estimated fetal weight (EFW) above the 95th percentile for gestational age. Amniotic fluid volume was within normal range, and the amnion–chorion interface appeared fully intact on all imaging planes. The patient was scheduled for reassessment at 36–37 weeks.

At 36 weeks and 4 days, repeat ultrasonography demonstrated fetal biometric parameters substantially advanced relative to gestational age: biparietal diameter (BPD) corresponded to 39 weeks and 1 day, head circumference (HC) to 37 weeks and 4 days, and femur length (FL) to 38 weeks and 3 days. AC measured 382 mm, with an EFW of 4,114 g, again exceeding the 95th percentile. On this examination, a freely mobile, multilayered membranous structure – exhibiting undulating motion with fetal movement and containing an echo-free interface consistent with interposed fluid – was identified beneath the anteriorly positioned placenta, distinct from the chorionic plate (Figure 1). A schematic timeline of the clinical course, including the progression from intact membrane architecture to CMS diagnosis and subsequent PPROM, is provided in Supplementary Figure 1. The separated membrane was most prominent in the region of the placental cord insertion, where the fetal upper extremities were also located (Figure 2).

**Figure 1:**
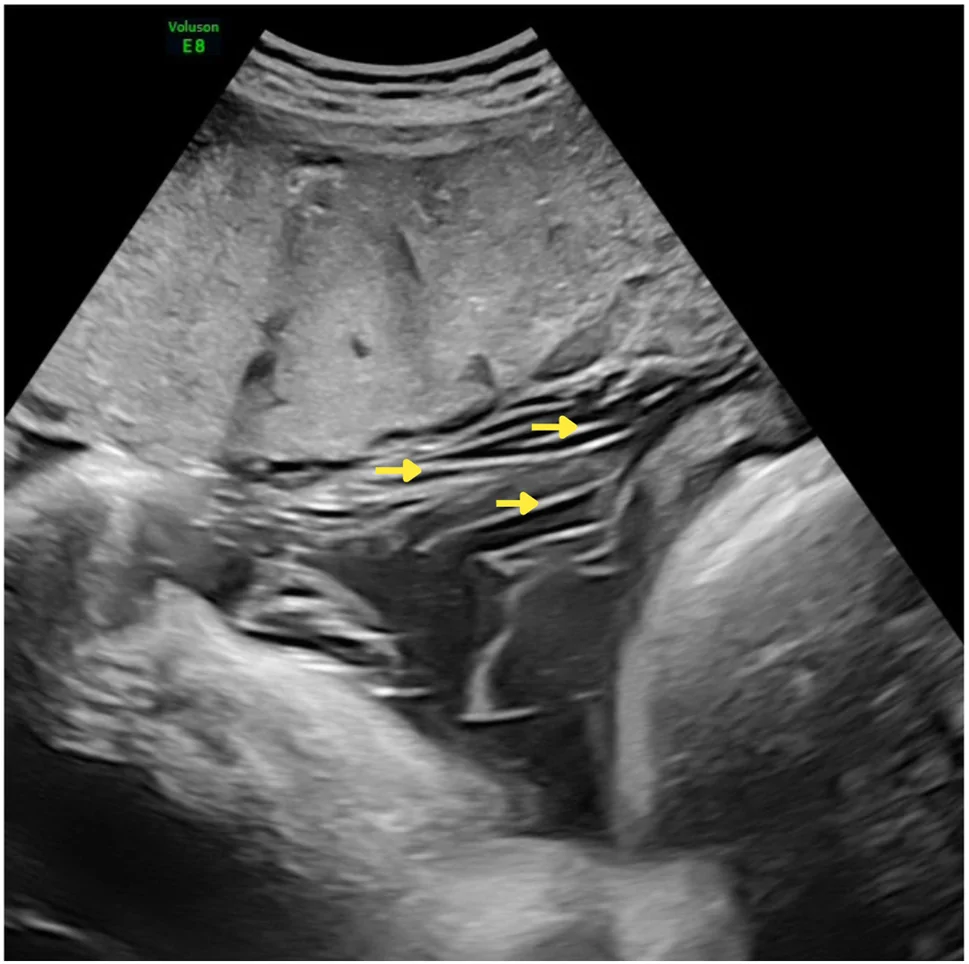
Ultrasonographic appearance of chorioamniotic membrane separation (CMS) at 36 weeks of gestation. Floating layers of the detached amniotic membrane are visualized beneath the anteriorly located placenta (yellow arrows).

**Figure 2:**
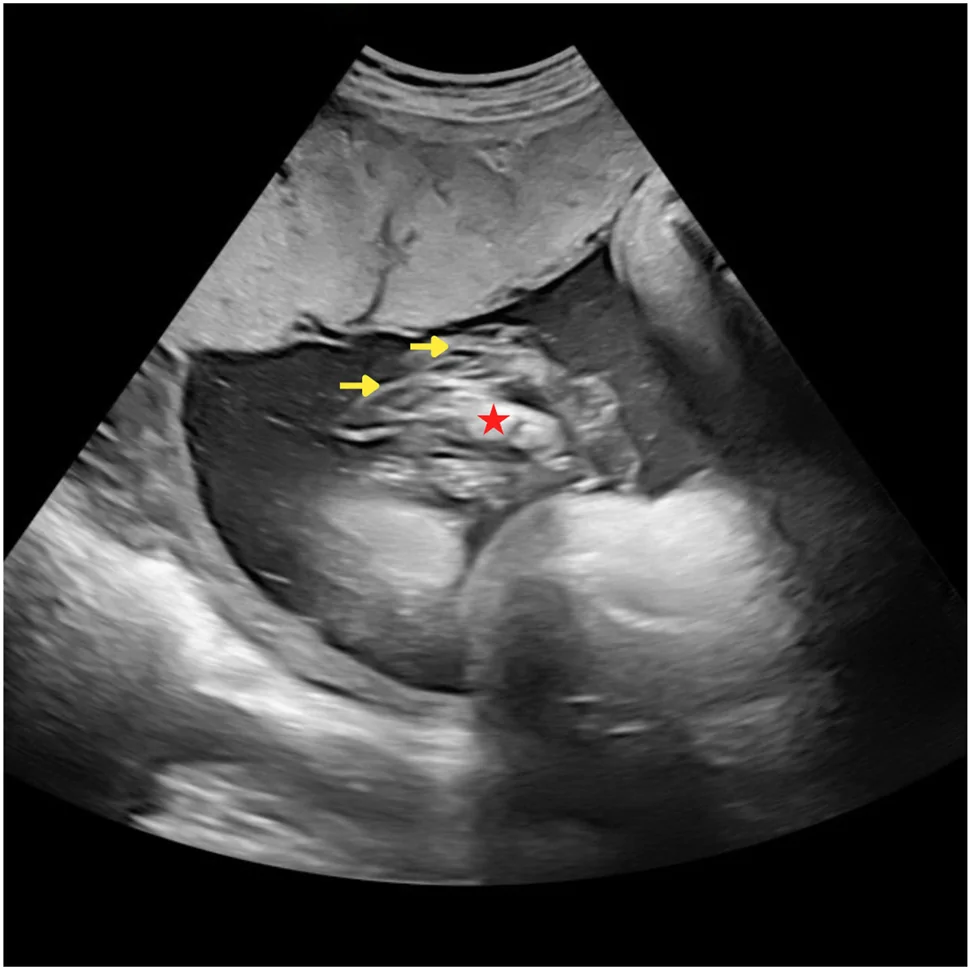
Evaluation of the relationship between the detached amniotic membrane and fetal extremities. The separated amniotic membrane folds (yellow arrows) are seen in close proximity to the fetal upper extremity, specifically the phalanges (red asterisk).

The sonographic appearance raised immediate concern for amniotic band sequence (ABS). Real-time ultrasonography demonstrating the relationship of the separated membranes to the fetal upper extremity and umbilical cord is provided in Supplementary Videos 1 and 2. All fetal extremities accordingly underwent systematic evaluation: anatomical continuity was preserved in each, and color Doppler ultrasonography confirmed unobstructed distal arterial perfusion in both upper limbs, with no evidence of constriction ring morphology or soft-tissue indentation. The absence of band-like structures with constrictive configuration, combined with intact extremity vascularity, supported CMS as the working diagnosis. The patient reported no history of invasive intrauterine procedures, physical trauma, or intercurrent infection; the condition was therefore classified as spontaneous in etiology. She was admitted for inpatient maternal–fetal surveillance.

Fetal heart rate monitoring remained reactive throughout the initial observation period. 12 hours after admission, preterm premature rupture of membranes (PPROM) occurred – representing a total interval of less than 24 h from diagnosis and less than one week from a previously normal sonographic examination. Given the gestational age of 36 weeks and 5 days and the presence of a prior uterine scar, delivery by cesarean section was performed. A live male infant weighing 4,170 g was delivered, with Apgar scores of 8 at 1 min and 9 at 5 min.

Intraoperative findings corroborated the prenatal diagnosis: upon entry into the uterine cavity, a freely floating amniotic membrane was visualized adjacent to the fetal presenting part (Figure 3). The intraoperative findings confirming chorioamniotic membrane separation are further demonstrated in Supplementary Video 3. Systematic dissection of the placenta and fetal membranes demonstrated near-complete amnion–chorion dissociation extending across the majority of the membrane surface (Figure 4). Histopathological examination was not performed. The postoperative course was uncomplicated for both mother and neonate.

**Figure 3:**
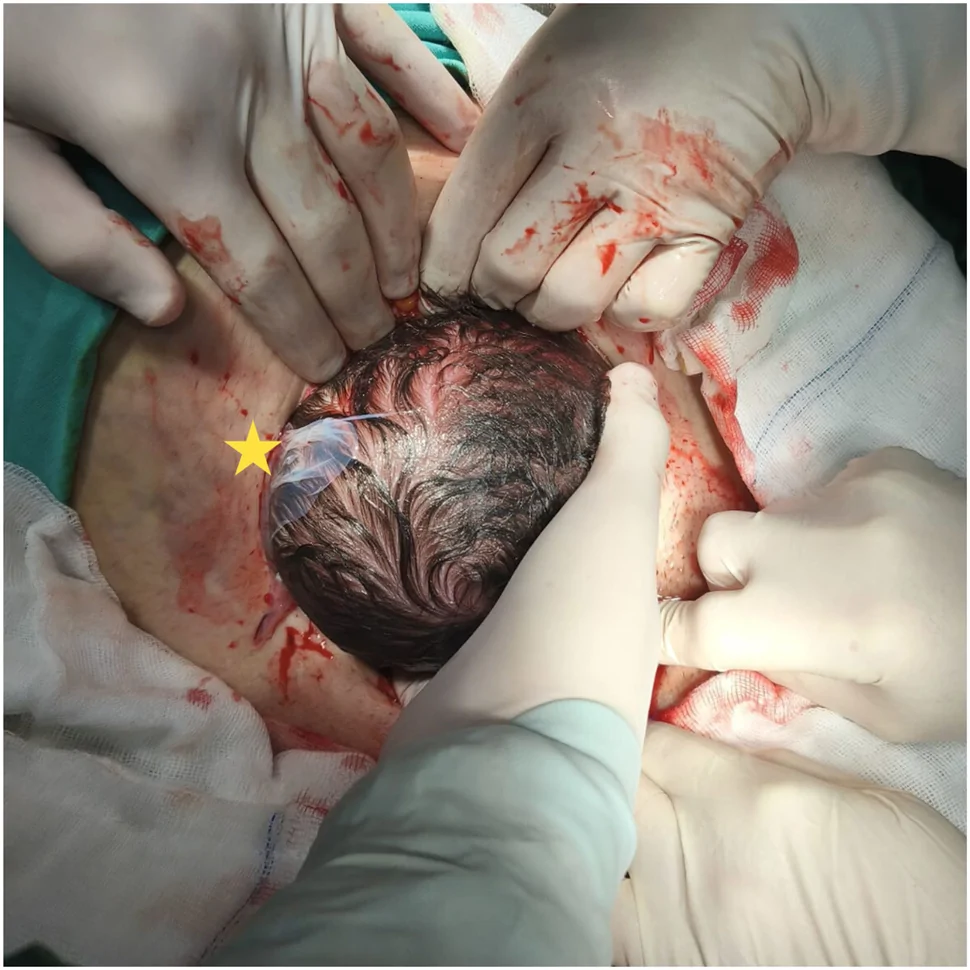
Intraoperative photograph obtained during cesarean section demonstrating the separated amniotic membrane adjacent to the fetal head, indicated by the yellow asterisk.

**Figure 4:**
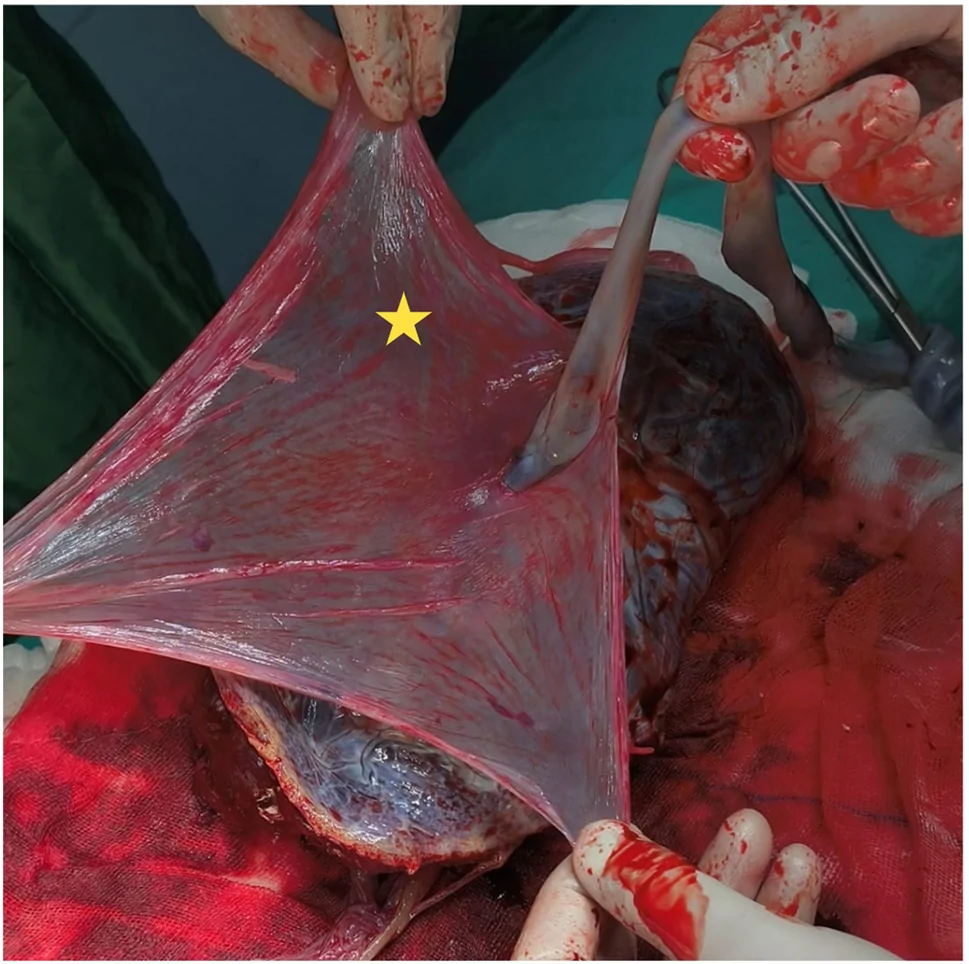
Photograph of the placenta and fetal membranes after delivery, demonstrating near-complete separation of the amniotic membrane from the chorion (yellow asterisk).

## Discussion

This case illustrates a clinically important presentation of spontaneous chorioamniotic membrane separation (CMS): acute onset in the third trimester, a sonographic appearance mimicking amniotic band sequence (ABS), and progression to preterm premature rupture of membranes (PPROM) within 12 h of diagnosis. Although CMS has been described in association with adverse perinatal outcomes, such a rapidly progressive clinical course in late gestation remains rarely documented [5], 8]. This observation suggests that late-onset CMS should not be considered a stable incidental finding, but rather a dynamic and potentially unstable condition with risk of abrupt deterioration.

Free-floating membrane structures in proximity to fetal extremities represent a key diagnostic challenge in prenatal ultrasonography. ABS carries a direct risk of soft-tissue constriction, digital amputation, severe structural deformity, and fetal demise [6]; and its sonographic overlap with CMS is well recognized [1], 3], 7]. However, the underlying pathophysiology differs fundamentally. Although amniotic band formation has been reported in a subset of spontaneous CMS cases [1], it is not invariable, and conflation of these diagnoses may lead to both unnecessary intervention and inadequate surveillance. In the present case, systematic anatomical assessment demonstrated preserved extremity integrity, and Doppler ultrasonography confirmed unobstructed distal perfusion without evidence of constrictive bands. This distinction avoided immediate intervention while justifying close inpatient surveillance. Importantly, accurate differentiation does not eliminate clinical concern, but instead redirects management toward vigilant monitoring and timely intervention when indicated.

The present case raises a hypothesis-generating observation: fetal macrosomia may be associated with a shortened latency period. However, this association is based on a single case and remains speculative; it should not be generalized until confirmed by larger case series or cohort studies. Once dissociated from the chorion, the amniotic membrane loses its principal structural scaffold and becomes more susceptible to tensile failure under mechanical load [5], 9], 10]. The coexistence of a prior uterine scar – a recognized predisposing factor for CMS development [11] – may have independently reduced membrane resilience. The compressed latency observed here suggests that in late-onset CMS, particularly when accompanied by potential mechanical risk factors, surveillance intensity and preparedness for acute intervention should account for the potential for rapid decompensation.

Spontaneous and iatrogenic CMS are frequently analyzed as a unified cohort, but accumulating evidence suggests that spontaneous cases carry a meaningfully more adverse clinical trajectory. The systematic review by Zhu et al. [3] reported preterm birth rates exceeding 50 % and perinatal mortality above 10 % in spontaneous CMS – figures substantially higher than the 4 % mortality reported by Bibbo et al. [2], whose series incorporated both spontaneous and iatrogenic cases, a methodological feature that may attenuate the mortality signal attributable to spontaneous cases alone.

The prognostic disparity likely reflects a systematic detection asymmetry: iatrogenic CMS is identified at the time of the inciting procedure, enabling immediate surveillance initiation, whereas spontaneous CMS is typically discovered only after a complication has already declared itself – as illustrated by Govaerts et al. [4] and Gerson et al. [8]. A recently reported case of complete spontaneous CMS detected at 35 weeks required cesarean delivery on the day of diagnosis due to acute fetal compromise [12]. The PPROM occurring within 12 h of diagnosis in our patient reinforces that late-onset spontaneous CMS may follow a rapidly progressive and unpredictable course. Accordingly, late-onset CMS should be approached as a time-sensitive condition, and even in the absence of immediate fetal compromise, consideration may be given to inpatient monitoring and preparedness for delivery.

This case illustrates that late-onset spontaneous CMS warrants risk stratification distinct from second-trimester or iatrogenic presentations. To our knowledge, this is among the first reports to suggest a possible association between fetal macrosomia and rapid progression in the CMS-to-PPROM interval; further systematic documentation is required to better define the clinical behavior and risk profile of late-onset CMS.

A limitation of this report is the absence of histopathological examination of the placenta and fetal membranes, which might have provided additional insight into the underlying mechanism of membrane separation.

## Clinical implications

Currently, there is no established guideline or standardized management protocol for spontaneous CMS diagnosed in the third trimester; clinical decisions in this setting are largely guided by individualized risk assessment and clinical judgment.

Late-onset spontaneous CMS detected in the third trimester may warrant consideration of inpatient surveillance rather than routine outpatient follow-up, particularly in the presence of potential mechanical risk factors such as fetal macrosomia or prior uterine scar. Careful sonographic differentiation from amniotic band sequence is essential to avoid unnecessary intervention while recognizing the risk of membrane instability. Given the potential for rapid progression, early delivery planning may be considered on an individualized basis, especially when diagnosis occurs at or beyond 36 weeks of gestation.

## Supplementary Material

Supplementary Material

Supplementary Material

Supplementary Material

Supplementary Material

Supplementary Material

Supplementary Material

Supplementary Material
