# Supplementary material for: Late-onset spontaneous chorioamniotic membrane separation mimicking amniotic band sequence with rapid progression to preterm premature rupture of membranes
Source: Case Rep Perinat Med. 2026 Oct 22;15(1):20260024. doi: 10.1515/crpm-2026-0024 (PMC13690055; doi:10.1515/crpm-2026-0024)
Supplement: Supplementary file 1 [file j_crpm-2026-0024_suppl_001.docx]

**Supplementary Material**

- Supplementary Figure 1: Schematic timeline of the clinical course, illustrating the interval from intact membrane architecture at 35 weeks to sonographic diagnosis of CMS at 36 weeks and 4 days, and subsequent PPROM within 24 hours of diagnosis
- Supplementary Video 1: Real-time ultrasonography demonstrating the separated amniotic membranes surrounding the fetal upper extremity
- Supplementary Video 2: Real-time ultrasonography demonstrating the separated amniotic membranes adjacent to the umbilical cord
- Supplementary Video 3: Intraoperative video recording confirming chorioamniotic membrane separation. Audio content was removed to ensure patient confidentiality. Written informed consent for publication was obtained.
